# Test of Motor Proficiency Second Edition (BOT-2) Short Form: A Systematic Review of Studies Conducted in Healthy Children

**DOI:** 10.3390/children8090787

**Published:** 2021-09-09

**Authors:** Danilo Radanović, Dušan Đorđević, Mima Stanković, Damir Pekas, Špela Bogataj, Nebojša Trajkovic

**Affiliations:** 1 Faculty of Sport and Physical Education, University of Novi Sad, 21000 Novi Sad, Serbia; radanilo17@gmail.com; 2 Faculty of Sport and Physical Education, University of Niš, 18000 Niš, Serbia; dusandjordjevic1995@gmail.com (D.Đ.); mima.stankovic974@gmail.com (M.S.); 3 Faculty of Kinesiology, University of Zagreb, 10110 Zagreb, Croatia; damir.pekas@kif.unizg.hr; 4 Faculty of Sport, University of Ljubljana, 1000 Ljubljana, Slovenia; spela.bogataj@kclj.si; 5 Department of Nephrology, University Medical Centre Ljubljana, 1000 Ljubljana, Slovenia

**Keywords:** assessment, BOT-2, healthy children, motor skills, motor efficiency

## Abstract

Motor skill competence of children is one of the important predictors of health because if a child is physically active during early childhood, the possibility of occurrence of many chronic diseases in adulthood will be reduced. The aim of this study was to systematically review the studies conducted in healthy children using the shorter form of the Bruininks-Oseretsky (BOT-2) and to determine the applicability in cross-sectional studies and pre-post designs. The search and analysis of the studies were done in accordance with the PRISMA guidelines. An electronic databases search (Google Scholar, PubMed, Mendeley, Science Direct, and Scopus) yielded 250 relevant studies conducted from 2011 to 2020. A total of 21 studies were included in quantitative synthesis, with a total of 3893 participants, both male and female. Through this study, the BOT-2 test proved its broad applicability, so it can be concluded that this test can be used to improve motor proficiency in a healthy population of children. Hence, it is necessary to invest a lot of time during the implementation of various programs so that children would adequately develop their basic motor skills so they broaden their own repertoire of movements.

## 1. Introduction

Basic motor skills are crucially important integral elements for mastering complex movements, but they are also necessary components for the building of movements required for involvement in sports [1], that consists of control over objects (throwing or catching the ball), fundamental locomotor movements (running, jumping, skipping), as well as of stability, i.e., balance [2]. The greatest development of basic motor skills occurs during early childhood and is associated with the physical [3] social and cognitive development of a child [4,5], as well as with many health factors, such as cardiorespiratory fitness [6], and adiposity [2,7]. According to Cattuzzo et al. [7], motor skill competence is the most important predictor of physical fitness that is related to health itself because if a child is physically active during early childhood, the possibility of occurrence of many chronic diseases in adulthood will be reduced [8].

Movement provides the ability to transfer information between the cerebral hemispheres, that is, the more a child moves, the more they learn and receive information from the environment [9]. This is a broad term that refers to the ability to exercise various motor skills in a consistent and very skillful way [10,11]. Every time a movement occurs, sensory-motor stimulation enables and helps children to interact and understand their environment, which largely depends on the body itself and the speed of processing perceptual information [12].

Motor development can be characterized by qualitatively different periods in the lifespan. It consists of six major periods in the development of motor skill behavior, which are the reflexive, preadapted, fundamental patterns, context-specific, skillful, and compensation periods. These are not age-determined or empirically provable periods and they are meant to be a heuristic device to assist our conceptualization of the vast changes that occur in movement and mobility across the lifespan [13]. Stodden et al. and Clark agreed that motor skills cannot develop naturally with age due to the influence of various environmental factors [1,2]. Such outside influences result in a divergence of growth and development and one of the most common sedentary activities that impede the development of children’s motor skills is the use of tablets or screen time [14,15]. In that regard, there are also socioeconomic status, level of education of parents, the interaction between family members or siblings, since being an only child could have implications on child development. Siblings are an essential component of family systems and play a significant role in learning and development [16,17]. Having the benefit from an older sibling leads to faster development for the second-born child as well [18,19]. There are also schooling andsocio-cultural context to be considered [20], and if children are provided with opportunities to properly broaden their repertoire of skills, thusly, motor efficiency and quality of movement shall also improve [21]. Sutopo et al. [22] have also found that there was difference in the effect of the type of birth on motor ability in early childhood, where children born by cesarean delivery have highest motor ability, then the normal birth children and new birth using pacemaker.

It is a critical issue in terms of motor skills for children, in which largely depends on their motor experience [23], and the negative environmental impact may reduce their ability to participate in physical activities later in life [24]. In that case, the key thing for parents is to ensure that a child partakes in everyday activities, where participation itself is a key component of the overall health status of a child [25]. Many authors state the importance of daily physical activities of children [26,27,28,29], where manipulation of objects along with bilateral coordination improves in preschool age [30,31]. More precisely, during the third and fourth year of life, a child first learns to button up so that during the fourth and fifth year of life, they can learn to tie laces on their own [32].

There are numerous studies in which participants were with some mental or physical disability, and they used mentioned test for the evaluation of motor proficiency. According to the above-given facts, motor proficiency in the population of healthy children is in lacking due to the negative social factors, different cultural aspects, and socioeconomic status. Hence, the short form of the BOT-2 test is designed for children with different impairments, and there are no review studies that systematize findings of research conducted by the BOT-2 test where the participants were the healthy population of children. Based on the given facts, the aim of this study was to systematically review the studies conducted in healthy children using a shorter form of the Bruininks-Oseretsky (BOT-2) and to determine the feasibility in cross-sectional studies and pre-post designs. The main focus of this paper is to put this test in advantage, so that even a healthy population of children should undergo it. Also, the importance of the study lies in promoting motor competence, since the influence of the already known external risk factors, as well as promoting natural growth and health.

## 2. Materials and Methods

### 2.1. Description of the Testing Tools

The Bruininks-Oseretsky test [33], or the so-called BOT-2 test, has been designed to assess fine and gross motor skills (motor skill efficiency) of children 4 through 21 years of age. This test has a very broad application in physical therapy, adaptive physical exercise, and even in science. It is used to monitor motor efficiency by assessing motor interventions, but also by helping decision-making in order to adapt various programs for children [34]. The shorter form of the test consists of fourteen subtests, which assess fine motor precision and integration, bilateral coordination, balance, running speed, agility, upper body coordination, and strength. The reliability of all the mentioned items is above 0.70. It should be noted that the test validity and reliability were proved in the sample of healthy children [35,36,37]. The shorter form of the test consists of fourteen subtests, which assess five tests of motor skills (Drawing, Lines through Paths-Crooked, Folding Paper, copying a Square, Copying a Star, Transferring Pennies) and nine gross motor skills (Jumping in Place-Same sides Synchronized, Tapping Feet and Fingers-Same sides Synchronized, Walking Forward on a Line, Standing on One Leg a Balance Beam-Eyes Open, One-Legged Stationary Hop, Dropping and Catching a Ball-Both Hands, Dribbling a Ball-Alternating Hands, Knee Push-ups, Sit-ups).

In the BOT-2 test, the raw scores have to be converted. Initial test results are converted into points ranging from 2 to 13, and then by adding all the points, we obtain the total result. Normative data are given in the BOT-2 test manual, which can also be used for the standardization of results and percentile ranking. For example, in the item Transferring Pennies, the participants are given two 15-s periods to toss as many pennies as they can into a receptacle. If nine coins are tossed into the receptacle in the first trial and ten in the second, then the final score is 9–10, and on the comparison, table the score is 4. BOT-2 test materials and equipment include manual, examinee booklets, blocks, cards, pennies, red pencils, shuttle block, target, Administration easel, record forms, balance beam, box, knee pad, penny pad, scissors, string, tennis ball, stopwatch, a tape measure, a table, and two chairs. The time required for conducting this test is approximately twenty minutes andthe result of the examinee is recorded on the evaluation form.

### 2.2. Literature Review

The search and analysis of the studies were done in accordance with the PRISMA (Preferred Reporting Items for Systematic Reviews and Meta-analyses) guidelines [38] and gained Systematic Review registration number: 270495. The research included studies conducted from 2011 to 2020, and the literature relevant for this type of research, available with the following databases: Google Scholar, PubMed, Mendeley, Science Direct and Scopus. The following keywords were used during the databases search: assessment, BOT-2, healthy children, motor skills, motor efficiency.

The obtained data were analyzed using a descriptive method, and all titles and abstracts were scoured for potential papers covered by this systematic review, while, in order to increase search sensitivity, the research strategy was modified and adapted to each database and inquiry. If following a detailed assessment, the obtained studies met the inclusion criteria they were accordingly considered to be relevant to this study.

The search for studies, assessment of their value, and data extraction were conducted independently by two authors (D.Đ. and M.S.), and the lists of references from previously assessed and original research were also reviewed. After that, each author cross-examined the found works, which were then taken for further analysis or rejected.

### 2.3. Inclusion Criteria

The eligibility criteria included the studies published from 2011 to 2020, studies published in English, studies that included both genders where the participants were not older than twelve, studies wherein participants sample were healthy children, and the studies that have applied a shorter form of the BOT-2 test [33].

### 2.4. Exclusion Criteria

The exclusion criteria included the studies conducted before 2011, studies written in a language other than English, studies with participants over twelve years, obese children, and children with mental disorders, paralysis, autism, or Down syndrome.

### 2.5. Risk of Bias Assessment

The risk of bias was assessed according to the PRISMA 19 guidelines, that is, using the PEDro scale [39] to determine the quality of reviewed studies and the potential risk of bias (Table 1 and Table 2). Two independent reviewers assessed the quality and the risk of bias using checklists. Concordance between reviewers was estimated using k-statistics data to review the full text and assess relativity and risk of bias. In case of discordance as to findings of the risk of bias assessment, the obtained data was assessed by the third reviewer, who also gave the final decision. The k-rate of concordance between reviewers’ findings was k = 0.94.

### 2.6. Data Extraction

The data were extracted by two authors (D.Đ. and M.S.) independently, and a cross-examination defined whether the data were adequate. Then, the data were extracted and moved to an excel spreadsheet. Cochrane Consumer and Communication Review Group’s standardized data extraction protocol [61] was applied to extract study characteristics, including authors and year of study, information such as sample size, age, and types of an experimental program, duration, frequency and study results.

## 3. Results

### 3.1. Quality of the Studies

Of the total number of studies that were included in the quantitative analysis, eleven studies were cross-sectional in nature, while ten were pre-post design. Of all the cross-sectional studies included, 3 studies showed fair quality, 7 studies showed good, while only one study showed excellent quality. As far as longitudinal studies included, 2 studies showed fair quality, 7 studies showed good, while only one study showed excellent quality.

### 3.2. Selection and Characteristics of Studies

**Figure 1:**
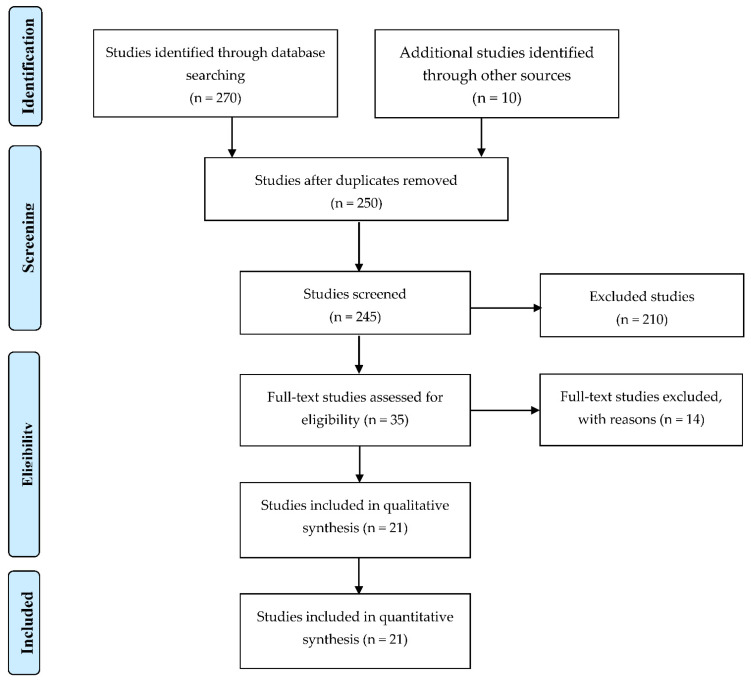
Collecting adequate studies on the basis of pre-defined criteria (PEDro flow).

A search of electronic databases and scanning of reference lists of studies revealed 250 relevant studies. Following the review of the titles or abstracts, 122 studies were rejected. Thirty-five full-texts were taken into consideration, and twenty-one were included in qualitative analysis (Figure 1). This study included a total of 3893 participants (3118 participants in cross-sectional and 775 in pre-post design studies).

Differences between various age categories were assessed by two studies [40,43], while gender differences were assessed in three studies [33,37,40], along with the assessment of the difference between the two nations [47]. Combined training was evaluated by one study [58], while gymnastics programs were evaluated by two studies [51,55]. The influence and correlation of modern technologies with motor skill efficiency of children was assessed by three studies [45,49,56], while the connection and influence of cognitive skills were assessed by four studies [41,44,53,54], and the relation and influence on academic achievements were assessed in as many as six studies [45,46,48,52,59,60]. Table 3 and Table 4 show in more detail the studies that have met the set conditions and were included in the quantitative analysis.

## 4. Discussion

The aim of this study was to systematically review the studies conducted in healthy children using a shorter form of the Bruininks-Oseretsky (BOT-2) and to determine the feasibility of cross-sectional studies and pre-post designs. It should be noted that the test validity and reliability were proved in the sample of healthy children [35,36,37].

The main finding of this systematic review is the fact that BOT-2 is adequate choise for monitoring motor competence in healthy children. Additionaly, studies that used BOT-2 for measuring motor competence showed that it is increasing throught early childhood as well as fact that girls outperform boys in the variables of fine motor skills, while boys outperform girls when it comes to the variables of gross motor skills [42,50,51,57]. When the variable of age is calculated, only first-grade and second-grade participants have shown expected results, compared to normative data [40,43]. Low socioeconomic status negatively affects motor skills efficiency [54], body mass index is not a significant factor when it comes to the correlation of children’s participation in sports and motor skill efficiency [48], and children that frequently use modern technology have shown a deficit in visual perception and motor skills [49,56]. Also, combined exercise gives much better results in motor skill efficiency variables compared to individual exercise [58], and it can be said that gymnastic programs produce a high level of effectiveness in the development of motor skills [51,55], while literacy, readability of handwriting and calculation skills depend a lot on the competence of motor skills [46,59,60], but also on cultural differentiation [62].

The studies by Gaul [40] and Gaul & Issartel [43] differ in that the participants in the first study were first graders, in the second study they were second graders, and both age categories were the only ones to achieve the expected results. Somewhat older participants in both studies did not show the expected results, which can be attributed to the fact that motor skills should develop gradually as children progress through primary school grades. The contrast in the results of slightly older children can be reflected in slower maturation [63]. Therefore the role of physical activities in schools and sedentary behavior needs to be reassessed.

Morley et al. [42] and Matarma et al. [50] attribute the fact that girls outperformed boys in fine motor skills and that boys were superior in gross motor skills, to outside physical activities, as well as to computer games. The environmental factor and the cultural factor play an important role here. Gabbard & Cacola [64] believe that girls have a greater tendency toward games that require skills such as jumping and skipping, while D’Hondt et al. [47] presume that boys have a greater affinity towards throwing or catching games. Laukkanen et al. [65] found that girls actualy can benefit from gross motor development, even if they were transitory in nature. In order to assess motor skill efficiency, the same authors also emphasize utilization of valid tests that cover a wide range of motor skills, as that way, deficiencies would be identified, and physical activities could be encouraged that would improve even with regard to different cultural backgrounds, as one’s society cultural values play an important role in the development of motor skills [66,67].

Through appropriate sports programs, children continue to integrate previously acquired motor skills into more complex systems of action [68], which leads to a gradual combining of abilities and their development into even more complex abilities, with all this occurring as a result of partaking in a number of sports activities or games [2]. Girls who have accomplished a program of combined physical exercises showed better results in motor skills efficiency compared to girls who practiced swimming or dancing. Swimmers are characterized by speed of movement and agility, while dancers show better flexibility, have better joint mobility and better body control [58]. Highly coordinated movements were the main requirements of combined exercise, therefore girls performed complex activities with much better result [69]. On the other hand, the multi-component form of sports activities provides an excellent foundation for motor skill development, supports long-term performance in sports, but it also builds later expertise and experience at the highest level [70].

Gymnastics programs can play a significant role in adequate growth and development. Some authors [71,72] consider that gymnastics programs can be one of the best tools for learning many fundamental movements and skills, and research shows that participation of children in gymnastics programs results in a significant improvement of motor skills [73,74,75], shows benefits in skeletal development [76], and social behavior [77]. Božanić et al. [51] and Karachle et al. [55] assessed the influence of conducting gymnastics program on the development of motor efficiency and fundamental movements, and in that regard, it should be noted that the subjects of both studies improved their motor skill efficiency and fundamental movements. Development of motor skills is further increased when children grow and develop their abilities in an environment that offers more opportunities to partake in development activities [51,74,75,78], and these factors may be potential indicators of good health and proper growth and development, as the performance of such sport activities allows better connectivity and economy of movement, improves strength and allows greater endurance capacity, with minimal effort invested for each movement [79].

The development of modern technologies, new inventions and discoveries greatly help in performing everyday tasks faster and easier. On the other hand, with the increased use of technological advantages and all of its benefits also has certain negative consequences, energy consumption consequently decreases and the potential danger is imminent if no action is taken in that regard in early childhood [80]. Children who do not often use modern technologies (tablets) show significantly better motor efficiency and visual perception than children who frequently use tablets [49]. Lin et al. [56] also proved this at the longitudinal scale; thus children who relied on tablets scored significantly lower in motor skill precision, fine motor integration, manual dexterity and grip strength, compared to the group of children that performed activities in a somewhat more traditional way, using scissors, threads and binding and by means of constructive games. These findings can affirm those of Cadoret et al. [57], which proved that children who spend more time behind the screen have a negative correlation of motor efficiency, that is, the time that a four-year-old spent in front of the screen manifested negatively in terms of motor efficiency, and even after three years, the same participants had shown the same negative correlation. The actions that are performed when using a tablet differ from those required for typical daily activities, and large differences in the use of fingers and hands have been observed in children who draw on paper compared to children who draw on a tablet device [81]. According to Ahearne et al. [82], that is not the case. They found that touch-screen platforms have many strengths, which differentiate them from other forms of media and offer the potential more positive effects. Interactive touch-screed applications offer a level of engagement not previously experienced with other forms of media and more akin to traditional play. They can also adapt to an individual child’s level, allowing complexity and providing positive feedback for a task. Despite the fact that in this modern age, children spend more time playing on tablets and applications instead of performing some more traditional activities such as assembling blocks, board games, puzzles, etc. [83,84], Dankert et al. [85] have found that children who apply those traditional physical activities while playing can improve motor skill efficiency and visual perception, while acquiring of motor skills through the application of modern technology remains to be subject of a future study.

Another method of gaining self-confidence and building a broad repertoire of movements and skills has its basis on psychomotor and cognitive health [86]. Kiphard [87] believes that it is possible to develop motor skills of children through focusing on the cognitive dimensions of movement, and Hernandez & Cacola [41] have found that there is a significant correlation between motor skill efficiency and cognitive abilities. It should be noted that as many as 60% of these four-year-olds had shown above-average physical coordination, given that 87% of them had previously attended a pre-school institution. Anna et al. [53] proved that a two-month psycho-motor skills program has a positive effect on motor skill efficiency and self-perception of pre-school children, regardless of the fact that the examinees of the experimental group scored lower on the initial assessment compared to the scores of the control group. These results can play a major role in improving the mentioned specific fields, especially fine motor skills, whereas the environment significantly influences motor behavior [88]. In that regard, the socioeconomic status of parents also needs to be considered, that is, a question needs to be raised whether parents can afford to to further enrich childrens’ knowledge through some additional educational content. Bradley & Corwyn [89] have already found that socioeconomic status is correlated to cognitive functions, but Shonkhoff et al. [90] have found that children whose parents are of higher standing engage in conversations more frequently, read more, and engage significantly more in societal activities, but also that they have shown better results in motor skills [91], compared to children whose parents are of somewhat lower standing, while Mulazimoglu-Balli [44] proved the opposite, that is, that children with lower socioeconomic status do not score that well in motor efficiency variables. Difficulties may emerge therein because by the end of the first grade, there is a lag in motor development and there is even a possibility that the lag may intensify in the upper grades of primary school if timely action is not taken. Timely intervention in that regard deems crucial [54], therefore it is necessary to introduce certain innovations to preschool institutions and programs, especially for children whose parents are dealing with such difficulties.

It can be said that the correlation between motor skills and academic achievement is still unclear, so the mechanisms of this connection are still being sought after [90]. Bornstein et al. [92,93] have found that babies who had shown better motor control and who explored their environment significantly more during that period showed better cognitive outcomes at four and ten, but they also had better academic achievements at ten and fourteen. Children with better motor skills show a higher percentage of intelligence and academic achievement [45], with a positive correlation between motor skills and writing, reading and pronunciation abilities and gross motor skills, bilateral coordination, balance and coordination of the upper extremities [60], while Seo [46] found a connection between motor skills and readability of writing, and positive correlation was found between motor skills and holding of objects with the readability of writing [94]. Spelling, directly related to the knowledge of a writing, requires good physical coordination [95], and pronunciation requires letter recognition and visual representation of that letter, which is related to memory and capacity of memory. Seo [46] also believes that children who show poor skill in holding of objects have poor readability in writing, although through the exercise of fine motor skills, children can acquire more developed and subtle motor precision as well as skills of manual manipulation, which can result in more precise and more controlled writing abilities. A physical exercise program could improve the motor skills of a child, but also the ability to calculate at an earlier age. Increased participation of children in activities that require cognitive-motor skills will also improve the above abilities [59]. This may have a direct effect, whereby fine motor coordination supports the use of mathematical manipulations often applied for teaching mathematics to pre-school children, but it may also have an indirect effect, whereby motor coordination skills improve the function of realization, which is correlated with fitness for school and pre-academic skills [96]. It is therefore of utmost importance that children who have shown difficulties in mastering motor skills during kindergarten are fully supported by teachers in order to adequately prepare for first grade [97].

Although from the scientific point of view all these matters are duly supported by research and design, there is very little evidence to the quality of work of teachers [98,99,100], who are supposed to be skilled and knowledgeable as to designing and implementing adequate programs intended for children. Based on this fact, school administration plays an important role in encouraging teachers to get acquainted with, implement, and maintain the continuity of planned activities. When a child starts their first grade of school, it is necessary to ensure that continuous physical activity in the new environment resumes or that a child even starts partaking in sports [52]. Since Ferreira et al. [48] determined that there is a connection between motor skills and engaging in sports, it is therefore important to build an adequate level of motor coordination already during childhood, which should result in greater abilities for future participation in sports, because motor coordination is the basis for the development of basic motor skills (running, catching) and it, in turn, influences the development of more pronounced skills required in sports, and eventually it plays a part in recognition of sports talents [101].

As far as comparison with some other tests for measuring motor proficiency, Venetsanou et al. [102] have found that the short form of BOT-2 test significantly overestimates the standard score compared to the longer form in a healthy population of children (mean age was 5.2 years). Holicky [103] came to the same conclusion, in the same population but different ages (mean age was 11.83 years). Khodaverdi et al. [104] conducted a correlation between the TGMD-3, the KTK test and the short form of BOT-2 test for motor competence in healthy girls (7–10 years old), where they found weak to moderate results. The same authors are also suggesting that KTK and short form of BOT-2 test may measure the same facets of motor competence and both TGMD-3 and short form of BOT-2 test composite measure different ones.

The drawback of this study may lie in the fact that the author did not have complete access to all databases, the authors decided to summarize all studies under the same analysis set. As for suggestions for further research, it would be examining the long form of the test, as well as a different sample of participants, that is, its aim should be to determine the applicability if the participants are children with some specifically clarified mental disability.

The conclusion drawn from the above facts would be that the improvement of motor skill efficiency depends on many individual motor and cognitive abilities, personal and situational factors, morphological characteristics and the environment from which a child obtains information. The main recommendation as to proper development of motor skills is to take action as early as possible, especially with regard to children whose parents are of somewhat lower standing, while also having in mind the relation with family and teachers at school as the crucial element for normal and natural growth and development of fundamental motor skills of children. It is necessary to invest a lot of time during the implementation of various programs so that children would adequately develop their basic motor skills, but also so that they broaden their own repertoire of movements, and it is necessary to perform periodic monitoring with a specified test in order to identify any shortcomings of a program and to rectify them.

Through this study, and from various perspectives, the BOT-2 test proved its broad feasibility and it may be noted that its main characteristics are simplicity of its conducting and the fact that it was designed in the form of a game. So it can be concluded that this test can be used to improve motor proficiency in healthy children.

## Figures and Tables

**Table 1:** PEDro scale for cross-sectional studies.

	Criterion											
Studies	1	2	3	4	5	6	7	8	9	10	11	∑
Gaul (2014) [40]	Y	N	N	N	N	N	N	Y	Y	Y	Y	4
Hernandez et al. (2015) [41]	Y	Y	Y	Y	Y	Y	Y	Y	Y	Y	Y	10
Morley et al. (2015) [42]	Y	Y	Y	N	N	N	Y	Y	Y	Y	Y	7
Gaul et al. (2015) [43]	Y	Y	Y	N	N	N	N	Y	Y	Y	Y	6
Mulazimoglu-Bali et al. (2016) [44]	Y	Y	Y	Y	N	N	N	Y	Y	Y	Y	6
Cadoret et al. (2018) [45]	Y	N	N	Y	N	N	N	Y	Y	Y	Y	5
Seo (2018) [46]	Y	N	N	N	N	N	N	Y	Y	Y	Y	4
D’Hondt et al. (2019) [47]	Y	N	N	N	Y	Y	Y	Y	Y	Y	Y	7
Ferreira et al. (2019) [48]	Y	Y	N	Y	N	N	N	Y	Y	Y	Y	6
Lin (2019) [49]	Y	N	Y	N	N	Y	N	Y	Y	Y	Y	6
Matarma et al. (2020) [50]	Y	N	N	Y	N	N	N	Y	Y	Y	Y	7

Legend: Y—criterion is satisfied, N—criterion is not satisfied.

**Table 2:** PEDro scale for longitudinal studies.

	Criterion											
Studies	1	2	3	4	5	6	7	8	9	10	11	∑
Božanić et al. (2011) [51]	Y	N	Y	N	Y	N	N	Y	Y	Y	Y	6
Vidoni et al. (2014) [52]	Y	Y	N	Y	N	N	N	Y	Y	Y	Y	6
Anna et al. (2016) [53]	Y	Y	N	N	N	Y	Y	Y	Y	Y	Y	7
Bellows et al. (2017) [54]	Y	N	N	Y	N	N	N	Y	Y	Y	Y	5
Karachle et al. (2017) [55]	Y	N	Y	Y	Y	Y	Y	Y	Y	Y	Y	9
Lin et al. (2017) [56]	Y	N	N	N	N	Y	Y	Y	Y	Y	Y	6
Cadoret et al. (2018) [57]	Y	N	N	Y	N	N	N	Y	Y	Y	Y	5
Gallota et al. (2018) [58]	Y	N	N	N	Y	Y	Y	Y	Y	Y	Y	7
Hudson et al. (2020) [59]	Y	Y	N	Y	N	Y	Y	Y	Y	Y	Y	8
Botha et al. (2020) [60]	Y	Y	N	Y	N	N	N	Y	Y	Y	Y	6

Legend: Y—criterion is satisfied, N—criterion is not satisfied.

**Table 3:** Cross-sectional studies of short form BOT-2 test.

First Author and Year of Publication	Aim of Study	Participants		Results
		Number and Groups	Years	
Gaul (2014) [40]	Evaluation of FMS, SMA, CL and AS	N-139	6–12	1st grade participants—expected results, 3rd an 5th grade participants—results below normative values
Hernandez et al. (2015) [41]	Correlation of MP and CA	N-32 M-15 F-17	4	+** MP and CA
Morley et al. (2015) [42]	Evaluation of MP depending on gender and SEs	N-369 M-193 F-176	4–7	F > M at FMS M > F at GMS
Gaul et al. (2016) [43]	Evaluation of FMS	N-253 M-139 F-114	6–12	Only 2nd grade participants showed expected results
Mulazimoglu-Bali et al. (2016) [44]	Correlation MP and BMI, as well as differences SEs in MP and BMI	N-60M-26F-34	6 ± 3.75	Lower SEs shows weaker MP, without +** MP and BMI
Cadoret et al. (2018) [45]	Correlation of MP and Aa, by means of CA	N-152	7	MP, Aa and CA +**
Seo (2018) [46]	Correlation FMS at handwriting legibility	N-52 M-23 F-29	5.76	+** FMS and handwriting legibility
D’Hondt et al. (2019) [47]	Evaluation and comparison of FMS i GMS	N-570 E_1_-325 E_2_-245	5.16	Similar results between groups, difference between two variables in favor of E_1_ because of the different PA
Ferreira et al. (2019) [48]	Correlation MP and participation in sports, as well as the role of BMI	N-707 M-332 F-375	6–10	+** participation in sports and MP, BMI isn’t relevant factor
Lin (2019) [49]	Difference between VP and FMS of children who use and who do not use applications on T	N-72 E_1_-36 E_2_-36	5.13	*** difference between groups in favor of E_2_, in VP and FMs
Matarma et al. (2020) [50]	Gender differences at MP, influence PA and family at MP	N-712	5	*** difference (8 tests) F > M, without *** PA and family at MP

Legend: N—total number of participants, M—male, F—female, E—experimental group, MP—motor proficiency, FMS—fine motor skills, GMS—gross motor skills, SMA—sensory motor abilities, CL—coordination level, AS—auditory stimuli, CA—cognitive abilities, SEs—socioeconomic status, BMI—body mass index, Aa—academic achievement, VP—visual perception, T—tablet, PA—physical activity, +**—positive correlation, ***—statistical significance.

**Table 4:** Effects of different programs on short form BOT-2.

First Author and Year of Publication	Aim of Study	Participants		Exercise Program	Results
		Number and Groups	Years		
Božanić et al. (2011) [51]	Gender difference in acquiring basic Mp under the influence of GP	N-58 M-34 F-24	6 ± 0.5	10 weeks 3× a week 35 min	Differences in 2 variables in favor of F
Vidoni et al. (2014) [52]	Evaluation of MP	N-33 E-18 K-15	3.9–5	11 weeks 7× a week 30 min	E and K improved MP E ***
Anna et al. (2016) [53]	Effects of 8 week PM program on MP and SP	N-29 E-14 K-15	3.5–5	8 weeks 2× a week 40 min	E *** in MP
Bellows et al. (2017) [54]	Status MP at participants with low SEs and evaluation of effect of MMp program at MP	N-250 E-143 K-107	3–5	18 weeks 4× a week 15–20 min	*** effects E in MP except in the case of 3 variables
Karachle et al. (2017) [55]	Effects of 6 months RG program on MP	N-37 E-21 K-13	3–7	6 months 2× a week 90 min	E and K improved MP *** E
Lin et al. (2017) [56]	Effects of using T on development FMS	N-80 E_1_-40 E_2_-40	5	24 weeks 7× a week 20 min	E_2_ *** in regards to E_1_
Cadoret et al. (2018) [57]	Correlation of TFS and MP	N-113	4–7	3 years	TFS increases with age −** and effects on MP at all ages
Galotta et al. (2018) [58]	Influence of 3 different pre-sport program on MP	F-25 E_1_-10 E_2_-6 E_3_-9	4–6	4 months	E_1_(combined PA) is more effective than E_2_ (dance program) and E_3_ (swimming program)
Hudson et al. (2020) [59]	Correlation of CA-MP-PA with MP and early numeracy skills	N-53 E-27 K-26	3–5	8 weeks 2× a week	E *** in all monitored variables
Botha et al. (2020) [60]	Effects of SP-MP program and correlation GMS and LK	N-97	6–7	12 weeks 2× a week 60 min	SP-MP program *** FMS and GMS +** MP and LK

Legend: N—total number of participants, M—male, F—female, E—experimental group, K—control group, MP—motor proficiency, SP—self-perception, RG—recreational gymnastics, GP—gymnastics program, T—tablet, FMS—fine motor skills, GMS—gross motor skills, TFS—time in front of the screen, PM—psychomotor, MMp—Mighty Moves program; CA—cognitive abilities, LK—letter knowledge, PA—physical activity, ***—statistical significance, −**—negative correlation, +**—positive correlation.

## Data Availability

Not applicable.
